# Humans, machines, and double standards? The moral evaluation of the actions of autonomous vehicles, anthropomorphized autonomous vehicles, and human drivers in road-accident dilemmas

**DOI:** 10.3389/fpsyg.2022.1052729

**Published:** 2023-01-04

**Authors:** Maike M. Mayer, Axel Buchner, Raoul Bell

**Affiliations:** Department of Experimental Psychology, Heinrich Heine University Düsseldorf, Düsseldorf, Germany

**Keywords:** autonomous agents, autonomous vehicle, human driver, anthropomorphism, moral evaluation

## Abstract

A more critical evaluation of the actions of autonomous vehicles in comparison to those of human drivers in accident scenarios may complicate the introduction of autonomous vehicles into daily traffic. In two experiments, we tested whether the evaluation of actions in road-accident scenarios differs as a function of whether the actions were performed by human drivers or autonomous vehicles. Participants judged how morally adequate they found the actions of a non-anthropomorphized autonomous vehicle (Experiments 1 and 2), an anthropomorphized autonomous vehicle (Experiment 2), and a human driver (Experiments 1 and 2) in otherwise identical road-accident scenarios. The more lives were spared, the better the action was evaluated irrespective of the agent. However, regardless of the specific action that was chosen, the actions of the human driver were always considered more morally justifiable than the corresponding actions of the autonomous vehicle. The differences in the moral evaluations between the human driver and the autonomous vehicle were reduced, albeit not completely eliminated, when the autonomous vehicle was anthropomorphized (Experiment 2). Anthropomorphizing autonomous vehicles may thus influence the processes underlying moral judgments about the actions of autonomous vehicles such that the actions of anthropomorphized autonomous vehicles appear closer in moral justifiability to the actions of humans. The observed differences in the moral evaluation of the actions of human drivers and autonomous vehicles could cause a more critical public response to accidents involving autonomous vehicles compared to those involving human drivers which might be reduced by anthropomorphizing the autonomous vehicles.

## Introduction

In recent years differences in the cognitive processing of information about humans and animals in comparison to inanimate objects has gained increasing attention (Nairne et al., 2017). Whereas these differences had long been ignored in cognitive research, there has been a surge of interest in the prioritization of humans and animals over inanimate objects in memory and attention in recent years (New et al., 2007; Nairne et al., 2013; Altman et al., 2016; Popp and Serra, 2016; Komar et al., 2022). The cognitive mechanisms underlying these differences are hotly debated and remain yet to be identified (VanArsdall et al., 2017; Meinhardt et al., 2018; Popp and Serra, 2018; Bonin et al., 2022). Moral judgement is a domain in which it seems quite obvious to distinguish between humans and inanimate agents such as machines, drones, or artificial intelligence algorithms. At first glance, intuition may suggest that humans are held to a higher moral standard than machines which implies that the actions of humans should be judged more harshly than those of machines (*cf*. Li et al., 2016; Gill, 2020). However, the scientific literature on this issue is mixed. When differences were found, the actions of humans were often judged more leniently than those of machines (e.g., Young and Monroe, 2019). However, it has also been observed that the moral judgement of humans and machines depends on the type of decision that is made. For example, a plausible possibility is that it is specifically the self-sacrifice of a human that may be evaluated more favorably than that of a machine but not the sacrifice of others (*cf*. Sachdeva et al., 2015). Furthermore, it has been proposed that the actions of machines are more likely to be judged according to utilitarian standards than the actions of humans (Malle et al., 2015, 2016). The aim of the present study is to evaluate how robust the differences in the moral evaluation of humans and machines are by testing whether there are reliable differences in the evaluations of the actions of human drivers and autonomous vehicles in road-accident scenarios across conditions that differ in the degree to which they involve utilitarian action and self-sacrifice.

The question of how people judge the actions of autonomous vehicles in comparison to those of humans is of high applied relevance as well. Even though it may yet take several years of development until fully autonomous driving will have reached an acceptable level of safety and reliability (for analyses of accident reports with autonomous vehicles see, e.g., Favarò et al., 2017; Wang et al., 2020), autonomous-driving technology promises to bring many benefits eventually, such as less traffic congestion, potentially resulting in less pollution and reduced energy consumption (Bagloee et al., 2016). Autonomous vehicles may also open a new chapter in mobility-on-demand and car-sharing services that might reduce the individual and societal costs of mobility (Spieser et al., 2014). Once driving technologies will have reached an automation level that does not require humans to intervene, these technologies could increase the comfort of daily driving: Being freed of the driving task, passengers of autonomous vehicles could use the driving time for other activities (Anderson et al., 2016; Bagloee et al., 2016). Given that human error is a major cause of road accidents (National Highway Traffic Safety Administration, 2008), autonomous vehicles are also expected to increase traffic safety in the future (e.g., Anderson et al., 2016). However, accidents cannot be completely avoided. Apart from the fact that no technology will ever function without flaws (e.g., Lin, 2016; Gogoll and Müller, 2017), there is another reason why autonomous vehicles cannot avoid all accidents regardless of their driving performance: they share the roads with human road users whose behaviors are hard to predict (e.g., Lin, 2016; Koopman and Wagner, 2017; Nyholm, 2018).

Fatal accidents with autonomous vehicles can be expected to attract strong media attention during the first years of introducing automated driving technologies into daily traffic (e.g., Shariff et al., 2017; Jelinski et al., 2021). Two of the best-known examples of accidents involving vehicles with automated driving technologies are the 2016 Tesla accident and the 2018 Uber accident. In 2016, a Tesla Model S collided with a semitrailer, resulting in the death of the Tesla’s driver (National Transportation Safety Board, 2017). The Tesla accident likely represents the first fatal crash involving a vehicle with automated driving technologies (Yadron and Tynan, 2016). The 2018 Uber accident—in which an Uber vehicle struck and killed a pedestrian (National Transportation Safety Board, 2019)—might be the first fatal crash of a vehicle with automated driving technologies involving a non-motorized road user (Levin and Wong, 2018; Wakabayashi, 2018). The critical coverage of accidents in the media can negatively affect the public perception and acceptance of autonomous vehicles (Shariff et al., 2017; Anania et al., 2018). Currently, the public’s opinion on automated vehicles is mixed (Becker and Axhausen, 2017). Some studies indicate prevailing positive anticipation (Winkler et al., 2019) but others show more negative than positive emotions (Hassol et al., 2019; Tennant et al., 2019). People who are skeptical about using automated driving technologies often cite an unwillingness to yield control over the driving task to the autonomous vehicle as a reason for their skeptical attitude (Smith and Anderson, 2017; Winkler et al., 2019). The prospect of machines making decisions that might harm or kill humans might contribute to the discomfort of handing over the control of driving to autonomous vehicles (Li et al., 2016; Malle et al., 2016; Bigman and Gray, 2018). This widespread discomfort with autonomous vehicles making life-and-death decisions may—together with other unsolved problems such as legal issues—delay the adoption of automated driving technologies (e.g., Li et al., 2016).

Therefore, it is of interest to understand how people morally evaluate, in fatal accident scenarios, the actions of autonomous vehicles in comparison to those of human drivers. A large body of the literature is focused on the action that people think autonomous vehicles and humans ought to choose. What is considered the moral choice does not need to be identical for humans and autonomous vehicles. However, the results of several studies suggest that people want humans and machines to make similar choices in road-accident dilemmas (e.g., Bonnefon et al., 2016; Li et al., 2016; Kallioinen et al., 2019; Young and Monroe, 2019). Most of these studies are modeled after the Trolley Dilemma (Foot, 1967; Thomson, 1976, 1985) which can be used to assess moral preferences. In the Trolley Dilemma, a trolley is racing towards five people on the tracks. It is possible to divert the trolley to a sidetrack which will, however, result in the death of an unsuspecting track worker. Is it morally permissible to sacrifice one person to save five? Or should the trolley continue on its path and kill five people? According to utilitarianism, sacrificing one life to save many is morally justifiable based on the principle that decisions should minimize harm and death (e.g., Bentham, 1789; Mill, 2010) while deontology, which focuses on moral rights and duties (e.g., Kant, 2011), may declare the same action as impermissible as it violates the duty not to kill otherwise uninvolved people as a means to an end. A road-accident scenario with an autonomous vehicle fashioned after the trolley dilemma could be the following: An autonomous vehicle is about to crash into one or more pedestrian/s on the road. The only other option being left is to crash the vehicle into a road block which results in the death of the passenger of the autonomous vehicle. Even though there is some degree of variability in people’s preferences for the action of the autonomous vehicle in such a moral dilemma (e.g., Awad et al., 2018), one of the most pervasive preferences that have been identified is the utilitarian preference to minimize the number of deaths that result from the accident with the autonomous vehicle (e.g., Li et al., 2016; Awad et al., 2018; Kallioinen et al., 2019; Mayer et al., 2021).

In the present study we focus on the *moral evaluation* of the actions of autonomous vehicles and human drivers in accidents that have already occurred. Whether people evaluate the actions of autonomous vehicles and human drivers differently is a two-part question: First, is there a general cognitive tendency towards evaluating the actions of human drivers less critically than those of autonomous vehicles? Second, are the same moral principles applied to human drivers and autonomous vehicles to morally evaluate their actions? In several studies, Malle and colleagues have used different versions of trolley-type moral dilemmas to test whether people evaluate the actions of robots, artificial intelligence agents, and drones differently than those of humans (Malle et al., 2015, 2016, 2019). Interestingly, the results suggest that the moral evaluation of actions may differ between humans and machines. Specifically, the results of Malle et al. (2015) suggest that “robots are expected—and possibly obligated—to make utilitarian choices” (p. 122) and thus people “regarded the act of sacrificing one person in order to save four (a ‘utilitarian’ choice) as more permissible for a robot than for a human” (p. 122). There is also evidence indicating that people have a general tendency for blaming autonomous vehicles more harshly than human drivers for their actions in road-accident scenarios (Young and Monroe, 2019). If the latter result turns out to be a robust finding and people are more critical of the actions of autonomous vehicles than of the actions of human drivers, then the question arises as to whether anthropomorphizing autonomous vehicles (that is, assigning humanlike characteristics or properties to them; Epley et al., 2007; Bartneck et al., 2009) will help to shift the moral evaluation of autonomous vehicles closer to the moral evaluation of human drivers. Young and Monroe (2019) found evidence suggesting that describing the decision-making process of the autonomous vehicle in mentalistic terms (i.e., ascribing thoughts and feelings to the autonomous vehicle) may reduce the differences in blame between the autonomous vehicle and a human driver, and may make people’s responses to the autonomous vehicle’s decisions less negative. In a similar way, Malle et al. (2016) found that presenting a robot with a more human-like appearance reduced the differences in blame for the decisions of robots and humans in comparison to presenting a robot with a mechanical appearance.

The empirical evidence is thus as yet mixed. No overall difference in the evaluation of humans and machines has been found in some studies, but the evaluation may differ depending on whether the action conforms to utilitarian standards or not (Malle et al., 2015, 2016). In another study results have been found that are more in line with a general bias for judging human agents more favorably than machines (Young and Monroe, 2019). It also seems conceivable, if not plausible, that self-sacrifice may play a special role in the moral evaluation of humans and machines (Sachdeva et al., 2015). Specifically, a human driver who sacrifices their own life to spare the lives of others may be more morally praiseworthy than an autonomous vehicle that sacrifices the passenger whom it was designed to protect. These hypotheses were put to an empirical test in the present two experiments.

The primary aim was to test whether there are differences in the moral evaluation of actions of autonomous vehicles and human drivers in road-accident scenarios that differed in whether or not a self-sacrifice of the human driver was involved and in the degree to which utilitarian principles favored this option. Participants morally evaluated the actions of either a human driver or the actions of an autonomous vehicle. They were presented with road-accident scenarios in which the life of the person inside the vehicle was weighted against the lives of one, two, or five pedestrians. If there is a general *bias* toward evaluating humans more favorably than machines (Young and Monroe, 2019), then the actions of autonomous vehicles should be evaluated as more reprehensible and less morally justifiable than those of human drivers, irrespective of whether the action involves a self-sacrifice of the human driver and irrespective of the number of pedestrians on the road. However, a different hypothesis can be derived from the position that people are more likely to judge the actions of machines according to utilitarian standards in comparison to those of humans (Malle et al., 2015). If utilitarian actions are rated more favorably if the agent is a machine rather than a human, then the differences in the moral evaluation between human drivers and autonomous vehicles should crucially depend on the number of lives that can be saved by the action. Specifically, the moral evaluation should be biased in favor of the autonomous vehicle the more lives are spared and biased in favor of the human driver the more deaths are caused by the decision. Finally, based on the hypothesis that there is a special role of self-sacrifice in moral evaluations (Sachdeva et al., 2015), it can be hypothesized that the self-sacrifice of a human driver should be rated as more morally praiseworthy than the sacrifice of the person inside the vehicle by the autonomous vehicle. To anticipate, the results lend clear support to the hypothesis that the actions of the autonomous vehicle are evaluated as less morally justifiable and more reprehensible than those of the human driver. In Experiment 2 we tested whether this negative evaluation tendency can be reduced by anthropomorphizing the autonomous vehicle by assigning a first name (“Alina”) to it and by describing it in mentalizing terms (compare Waytz et al., 2014; Hong et al., 2020). The actions of the anthropomorphized autonomous vehicle were indeed evaluated more positively than the actions of the non-anthropomorphized autonomous vehicle which provides further support of the hypothesis that the difference in the moral evaluation of the actions of human drivers and autonomous vehicles can be reduced by assigning humanlike characteristics or properties to the autonomous vehicle (Young and Monroe, 2019). The hypotheses that the moral evaluation of the actions of human drivers versus those of autonomous vehicles may depend on the involvement of utilitarian standards and self-sacrifice received some initial support in Experiment 1 but the associated sample effect sizes were only small and Experiment 2 showed that these interactions were not reliable. We thus conclude that the dominant pattern is that of a general bias towards judging the actions of human drivers more favorably than those of autonomous vehicles.

## Experiment 1

### Methods

The experiment was conducted online using *SoSci Survey* (Leiner, 2019). In total, participation took about 10 min. Both experiments reported here were conducted in accordance with the Declaration of Helsinki. Informed consent was obtained from each participant before the experiment.

#### Participants

The sample was recruited via online advertisements. Undergraduate Psychology students received course credit for participating; other participants could enter a lottery to win a € 20 voucher for an online store. To be able to detect even small differences between the judgments of the actions of the human driver and the autonomous vehicle, valid data from 350 participants were necessary (see the next paragraph). Considering the typical data loss in online studies we continued data collection until the end of the week at which data from about 20 percent more than that figure were collected. Of the 444 participants who had started the study, 79 did not complete the experiment. In addition, five participants did not meet the a-priori defined inclusion criteria (being of legal age, having sufficient German language skills, and being able to read the text on screen according to self-reports). Valid data sets of 360 participants (266 women, 94 men), aged between 18 and 80 years (*M* = 27, *SD* = 11) were included in the analyses. Participants were randomly assigned to the human-driver condition (*n* = 187) or the autonomous-vehicle condition (*n* = 173).

We conducted a sensitivity power analysis with G*Power (Faul et al., 2007) in which we focused on the agent variable (human driver, autonomous vehicle; between-subjects) and on the action variable (sacrifice the pedestrian/s, sacrifice the person inside the vehicle; within-subject). Given a total sample size of *N* = 360, *α* = *β* = 0.05, and assuming a correlation of *ρ* = 0.20 between the levels of the action variable (estimated based on related results), small effects of about *f* = 0.15 (Cohen, 1988) could be detected for the agent variable. Note that due to the exclusion of the within-subjects number-of-pedestrians variable that was technically necessary to perform the analysis, the power analysis can only give an approximate indication of the sensitivity underlying this study.

#### Materials and procedure

First, participants read an introductory text. Depending on the assigned condition, the text stated that human drivers or autonomous vehicles have to handle different traffic situations, including inevitable accidents. The instructions were identical in both conditions, with the only exception that the instructions in the autonomous-vehicle condition included the definition of autonomous vehicles as fully self-driving cars capable of participating in traffic without the need of human intervention (see definition of level 5 driving automation, SAE International, 2021). Participants were then provided with an exemplary description of the accident scenarios they were asked to evaluate later in the experiment. The instructions in the human-driver condition read:

You will now see various traffic situations in which an accident with a vehicle is unavoidable.

In these situations, a person is driving along a road. Suddenly an obstacle and one or more people appear on the road. Neither timely braking nor evasive action is possible. This means that the driving person only has two options for action:

1. He/she drives into the obstacle. The person inside the vehicle dies.

2. He/she drives into the person or persons on the road who dies or die in the process, respectively.

Your task is to evaluate the action of the driving person in the presented traffic situations.

In the autonomous-vehicle condition, the instructions were identical, but “person” was replaced by “autonomous vehicle”.

**Figure 1:**
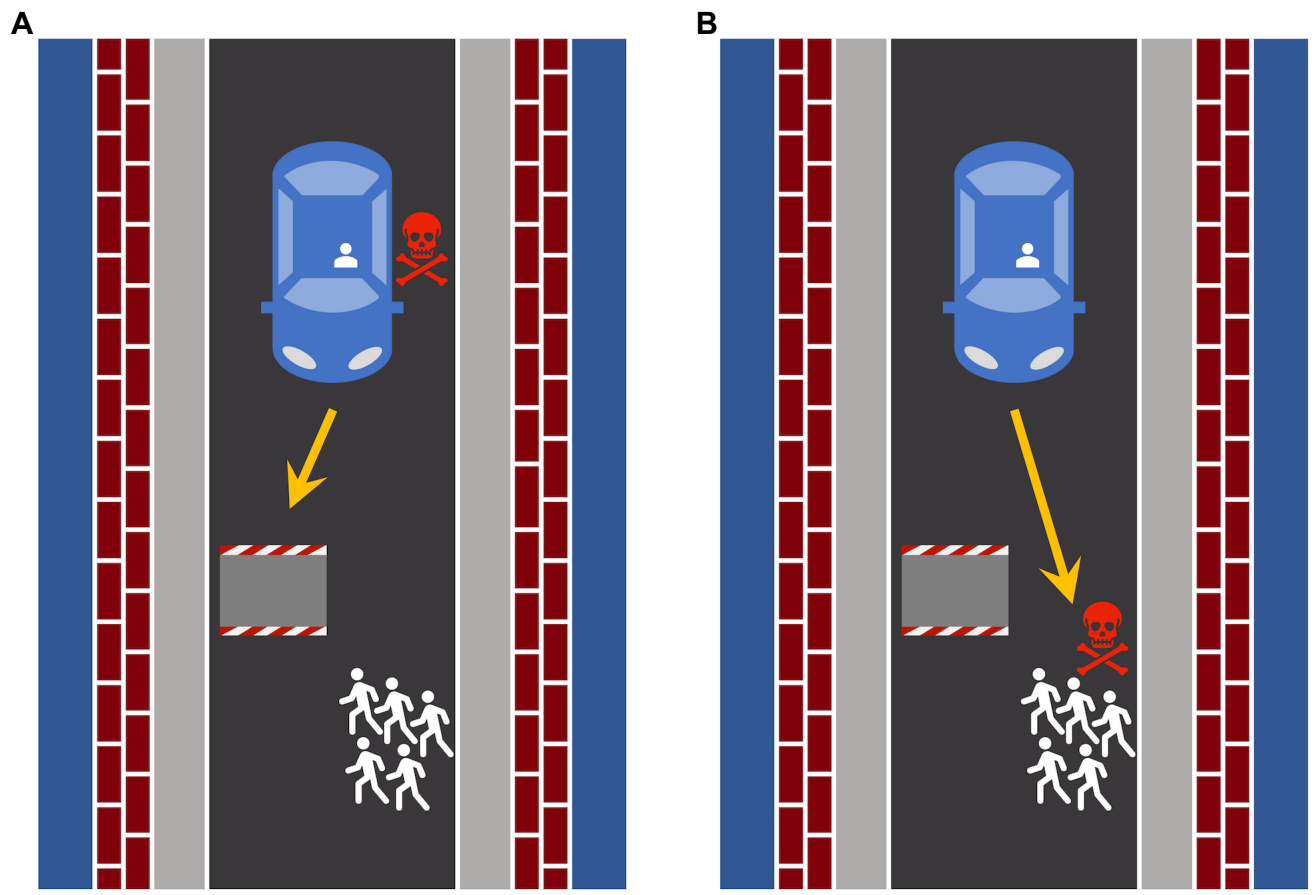
Two examples of the illustrations of the road-accident scenarios employed in the experiment. The images depict the two available actions for a road-accident scenario with five pedestrians on the road. **(A)** The person inside the vehicle is sacrificed to save the five pedestrians. **(B)** The five pedestrians are sacrificed to save the person inside the vehicle. The scenarios were created using Microsoft PowerPoint® and Apple Keynote®.

In each of the scenarios (see Figure 1 for an example), the agent (either a human driver or an autonomous vehicle) drove on a single-lane road and was suddenly confronted with an obstacle and at least one pedestrian on the road. As the agent could neither brake nor swerve, only two actions remained: The agent could either sacrifice the person inside the vehicle to save the pedestrian/s by crashing into the obstacle or sacrifice the pedestrian/s to save the person inside the vehicle. The scenarios were depicted as abstract sketches from a bird’s eye view. There were either one pedestrian, two pedestrians, or five pedestrians on the road. The agent had already taken one of the two available actions, represented by a yellow arrow. In each scenario, the agent either sacrificed the person inside the vehicle (Figure 1A) or the pedestrian/s (Figure 1B) who died because of the accident. The fatal consequence of the decision was illustrated by a red skull that was presented next to the person inside the vehicle or the pedestrian/s, depending on who was sacrificed. The visual depiction of the scenario was accompanied by a text vignette describing the situation, the action taken, and the action’s consequences. For example, if the autonomous vehicle sacrificed five pedestrians to save the person inside the vehicle, the text stated: “The autonomous vehicle drives into the persons on the street. The person inside the vehicle remains unharmed. The five persons on the street are killed.” Six different scenarios were obtained by combining two actions and three different numbers of pedestrians. The positions of the obstacle and the pedestrian/s (left or right side of the road) were counterbalanced. Altogether, four presentations of each of the six scenarios were presented, yielding 24 evaluations in total. The scenarios were presented in random order.

Below each image and the corresponding text vignette, participants were asked to evaluate the action (sacrifice the person inside the vehicle vs. sacrifice the pedestrian/s) of the agent (human driver vs. autonomous vehicle) from a moral perspective. The question repeated the agent, the action, and the action’s consequences for the two involved parties. For example, if the autonomous vehicle decided to sacrifice five pedestrians to save the person inside the vehicle, the question was: “How do you evaluate, from a moral point-of-view, the action of the autonomous vehicle to save the person inside the vehicle and to sacrifice the five persons on the street?”. Participants were asked to complete the sentence “From a moral point-of-view, I perceive the action as …” by choosing a category on a scale ranging from “very reprehensible” (1) to “very justifiable” (6). These labels were chosen based on a pilot study (*N* = 16) in which participants were asked to choose from six pairs of negative and positive labels the pair that best captured their moral evaluation of actions in road-accident dilemmas.

As an attention check at the end of the study, the participants were asked to indicate the type of agent that had been involved in the presented scenarios (“A human driver,” “An autonomous vehicle,” “I do not know”). As the statistical conclusions did not change in both experiments if participants who failed the attention check were included in the statistical analysis, we decided against the exclusion of data, following a recommendation of Elliott et al. (2022).

### Results

**Figure 2:**
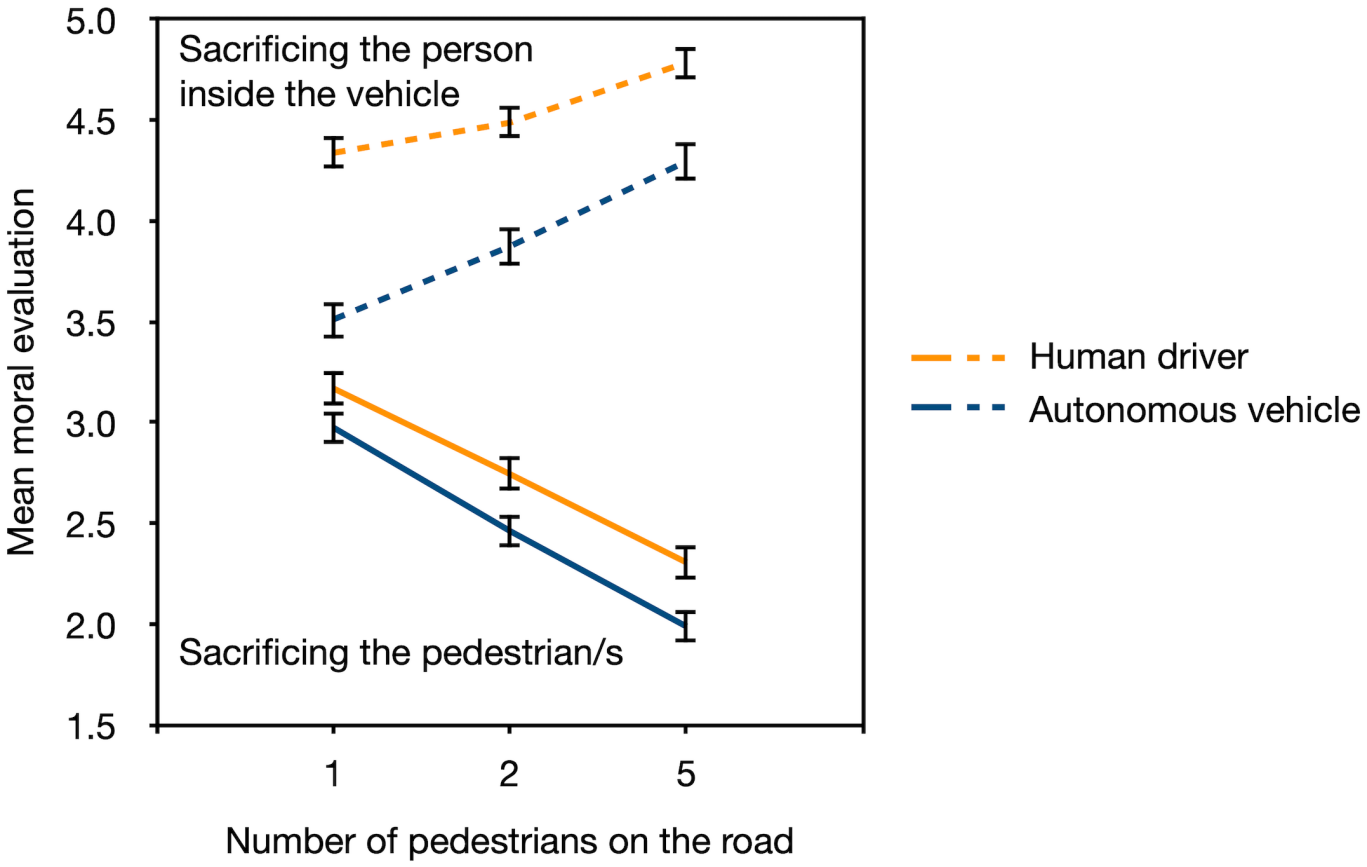
The mean moral evaluation of the actions (sacrificing the person inside the vehicle [dashed lines], sacrificing the pedestrian/s [solid lines]) as a function of the number of pedestrians on the road (1, 2, and 5) and the agent (human driver, autonomous vehicle). The moral-evaluation scale ranged from “very reprehensible” (1) to “very justifiable” (6). The error bars represent standard errors of the means.

In our analyses, we used the multivariate approach to repeated-measures analyses described, for instance, in a primer by O’Brien and Kaiser (1985). In contrast to the so-called univariate approach to repeated-measures analyses, the multivariate approach does not require the sphericity assumption to be met. This is a major advantage given that the sphericity assumption is violated in almost all repeated measures designs. Exact *F* statistics are reported. The 𝛼 level was set to 0.05 and all post-hoc comparisons were Bonferroni-Holm adjusted (Holm, 1979). The partial eta squared is used as a sample effect size measure. The mean moral evaluation of the actions as well as the standard errors of the means are depicted in Figure 2.

A 2 (agent: human driver, autonomous vehicle; between-subjects) × 2 (action: sacrifice the pedestrian/s, sacrifice the person inside the vehicle; within-subject) × 3 (number of pedestrians: one pedestrian, two pedestrians, five pedestrians; within-subject) analysis showed that the actions of the human driver (*M* = 3.64, *SE* = 0.05) were evaluated as more morally justifiable than the actions of the autonomous vehicle (*M* = 3.18, *SE* = 0.05), *F*(1,358) = 40.51, *p* < 0.001, *η_p_*^2^ = 0.10. Sacrificing the person inside the vehicle was evaluated more favorably (*M* = 4.23, *SE* = 0.06) than sacrificing the pedestrian/s (*M* = 2.61, *SE* = 0.06), *F*(1,358) = 340.82, *p* < 0.001, *η_p_*^2^ = 0.49. The interaction between agent and action was statistically significant as well, *F*(1,358) = 4.72, *p* = 0.030, *η_p_*^2^ = 0.01. Simple main effect analyses revealed that the human driver’s actions were always evaluated more favorably (*M* = 4.54, *SE* = 0.08, for sacrificing the person inside the vehicle; *M* = 2.74, *SE* = 0.08, for sacrificing the pedestrian/s) than those of the autonomous vehicle (*M* = 3.89, *SE* = 0.09, for sacrificing the person inside the vehicle; *M* = 2.48, *SE* = 0.08, for sacrificing the pedestrian/s), but the difference between agents was more pronounced for the decision to sacrifice the person inside the vehicle (*η_p_*^2^ = 0.08) than for the decision to sacrifice the pedestrian/s (*η_p_*^2^ = 0.02).

In addition, there was a significant main effect of the number of pedestrians on the road, *F*(2,357) = 23.94, *p* < 0.001, *η_p_*^2^ = 0.12 (*M* = 3.51, *SE* = 0.04, for one pedestrian; *M* = 3.40, *SE* = 0.04, for two pedestrians; *M* = 3.35, *SE* = 0.04, for five pedestrians). The direction of this effect, however, depended on the action that was taken, *F*(2,357) = 187.20, *p* < 0.001, *η_p_*^2^ = 0.51. An increase in the number of pedestrians led to a significant increase in the moral evaluation of sacrificing the person inside the vehicle (*M* = 3.94, *SE* = 0.06, for one pedestrian; *M* = 4.19, *SE* = 0.06, for two pedestrians; *M* = 4.55, *SE* = 0.06, for five pedestrians, respectively; all comparisons *p* < 0.001) while it led to a significant decrease in the moral evaluation of sacrificing the pedestrian/s (*M* = 3.08, *SE* = 0.06, for one pedestrian; *M* = 2.61, *SE* = 0.06, for two pedestrians; *M* = 2.15, *SE* = 0.06, for five pedestrians; all comparisons: *p* < 0.001). The effect of the number of pedestrians did not differ between agents, *F*(2,357) = 2.73, *p* = 0.067, *η_p_*^2^ = 0.02.

Finally, there was a significant three-way interaction, *F*(2,357) = 4.54, *p* = 0.011, *η_p_*^2^ = 0.02. We conducted a 2 (action: sacrifice the pedestrian/s, sacrifice the person inside the vehicle; within-subject) × 3 (number of pedestrians: one pedestrian, two pedestrians, five pedestrians; within-subject) repeated-measures analysis for each of the two agents separately. The action of sacrificing the person inside the vehicle was evaluated as significantly more morally justifiable than the action of sacrificing the pedestrian/s for both the human driver, *F*(1,186) = 241.01, *p* < 0.001, *η_p_*^2^ = 0.56, and the autonomous vehicle, *F*(1,172) = 117.40, *p* < 0.001, *η_p_*^2^ = 0.41. There was a significant main effect of the number of pedestrians on the road for both the human driver, *F*(2,185) = 18.70, *p* < 0.001, *η_p_*^2^ = 0.17, and the autonomous vehicle, *F*(2,171) = 6.79, *p* = 0.001, *η_p_*^2^ = 0.07. Finally, the interaction between action and the number of pedestrians on the road was significant for both the human driver, *F*(2,185) = 90.23, *p* < 0.001, *η_p_*^2^ = 0.49, and the autonomous vehicle, *F*(2,171) = 96.98, *p* < 0.001, *η_p_*^2^ = 0.53. When the person inside the vehicle was sacrificed, the action was evaluated as significantly more morally justifiable with an increasing number of pedestrians on the road for both the human driver (*M* = 4.34, *SE* = 0.08, for one pedestrian; *M* = 4.49, *SE* = 0.08, for two pedestrians; *M* = 4.79, *SE* = 0.08, for five pedestrians; all comparisons *p* < 0.001) and the autonomous vehicle (*M* = 3.51, *SE* = 0.09, for one pedestrian; *M* = 3.87, *SE* = 0.09, for two pedestrians; *M* = 4.30, *SE* = 0.10, for five pedestrians; all comparisons *p* < 0.001) while the reverse pattern emerged when the decision was to sacrifice the pedestrian/s for both the human driver (*M* = 3.17, *SE* = 0.09, for one pedestrian; *M* = 2.74, *SE* = 0.08, for two pedestrians; *M* = 2.31, *SE* = 0.08, for five pedestrians; all comparisons *p* < 0.001) and the autonomous vehicle (*M* = 2.98, *SE* = 0.09, for one pedestrian; *M* = 2.46, *SE* = 0.08, for two pedestrians; *M* = 1.99, *SE* = 0.08, for five pedestrians; all comparisons *p* < 0.001). The three-way interaction thus does not indicate that fundamentally different moral principles were applied to the evaluation of the actions of the human driver and to the evaluation of the actions of the autonomous vehicle, but the effect of the number of pedestrians on the moral evaluation of the action of sacrificing the person inside the vehicle was somewhat less pronounced for the human driver than for the autonomous vehicle.

### Discussion

The present study served to test whether there are differences in the moral evaluation of the actions of human drivers and autonomous vehicles. The most important finding is that the actions of the human driver were always evaluated as more morally justified than the actions of the autonomous vehicle, which suggests that there is a moral-evaluation bias in favor of the human driver.

Another aim of the present study was to evaluate whether the actions of human drivers are evaluated according to different moral principles than those of autonomous vehicles. Before addressing the qualitative differences in the moral evaluation of the human driver and the autonomous vehicle, we want to draw attention to the fact that there are striking similarities. Overall, the moral evaluations of the actions of the human driver and the autonomous vehicle depended on both the type of action that was evaluated (sacrificing the person inside the vehicle or the pedestrian/s) and the number of pedestrians on the road. Regardless of whether the actions of the human driver or the autonomous vehicle were evaluated, participants regarded actions that spared the maximum number of lives as more morally justifiable than other actions. The favorable evaluations of utilitarian actions are in line with demonstrations of overall preferences for utilitarian actions of human and machine agents in other studies (e.g., Li et al., 2016; Kallioinen et al., 2019). There was an interaction between agent and action, indicating that the decision of the human driver to self-sacrifice was evaluated more favorably than the action of the autonomous vehicle to sacrifice the person inside the vehicle, in line with a special role of self-sacrifice in moral judgement (Sachdeva et al., 2015). Furthermore, there was a three-way interaction between agent, action, and number of pedestrians, suggesting that the evaluation of the human driver’s decisions to sacrifice themselves was less dependent on the number of pedestrians on the road than the evaluation of the autonomous vehicle’s decisions to sacrifice the person inside the vehicle. At first glance, this finding is in line with the assumption that the moral evaluation of the actions of the autonomous vehicle depends more on utilitarian standards than the moral evaluation of the actions of the human driver. However, this finding can easily be explained by the fact that the decision of the human driver to self-sacrifice received favorable moral evaluations already when this meant sparing the life of only one pedestrian, and this favorable evaluation was hard to boost when more pedestrians were saved at the expense of the driver. It is also worth pointing out that the sample effect sizes of these interactions are quite small (the sample effect sizes of the two-way interaction between agent and action and the three-way interaction between agent, action, and number of pedestrians were *η_p_*^2^ = 0.01 and *η_p_*^2^ = 0.02, respectively). Therefore, it seems questionable whether interactions of such small magnitude can be robustly replicated in future experiments (see the Discussion of Experiment 2). Furthermore, the evaluations of the human driver’s actions were always more favorable than those of the autonomous vehicle irrespective of whether self-sacrifice or utilitarian actions were involved or not (*cf*. Figure 1). The dominant finding is thus that there is an overall bias towards a more favorable evaluations of the actions of the human driver over those of the autonomous vehicle.

Experiment 2 had two main aims. The first aim was to test whether the differences in the moral evaluations of the actions of the human driver and the autonomous vehicle found in Experiment 1 could be replicated. Due to the small sample effect sizes of the critical two-way and three-way interactions observed in Experiment 1, we thought it important to perform a high-powered replication before drawing any firm conclusions. Based on the sample effect sizes observed in Experiment 1, we expected that the main effect of agent—reflecting a more critical evaluation of the actions of the autonomous vehicle in comparison to those of the human driver—should also be obtained in Experiment 2 whereas it was questionable whether the two-way interaction between agent and action and the three-way interaction between agent, action, and number of pedestrians—that were both associated with small sample effect sizes—could be replicated. The second aim of Experiment 2 was to test whether anthropomorphizing the autonomous vehicle may help to narrow the gap between the moral evaluation of actions taken by an autonomous vehicle and a human driver in inevitable accidents with human fatalities. If the difference in the moral evaluation in the actions of the human driver and the autonomous vehicle is caused by some fundamental difference in the moral evaluation of humans and machines, anthropomorphizing the autonomous vehicle (that is, making it more similar to human agents) should reduce the differences in the moral evaluations.

## Experiment 2

### Methods

#### Participants

Participants were recruited from the online research panels of GapFish GmbH (Berlin, Germany). Of the 892 participants who started the study, 80 did not complete the experiment, 10 did not meet the *a-priori* defined inclusion criteria (being of legal age, having sufficient German language skills, and being able to read the text on screen according to self-reports), and 37 either withdrew their consent to the processing of their data or reported that not all pictures had been displayed during the study. Additionally, 10 participants were excluded due to double participation. The final sample consisted of 755 participants (317 women, 437 men, and 1 diverse), aged between 18 and 87 years (*M* = 46, *SD* = 15). Participants were randomly assigned to the human-driver condition (*n* = 248), the anthropomorphized-autonomous-vehicle condition (*n* = 250), or the autonomous-vehicle condition (*n* = 257).

Given the goal to test whether anthropomorphizing the autonomous vehicle would cause the moral evaluations of the autonomous vehicle to shift towards the more favorable evaluation of the human driver, it seemed important to increase the sensitivity of the statistical tests in Experiment 2. We decided to collect data from at least twice as many participants as in Experiment 1 and stopped data collection at the end of the week this criterion was surpassed. A sensitivity power analysis parallel to that conducted for Experiment 1 showed that, given a total sample size of *N* = 755 and otherwise identical assumptions, small effects of about *f* = 0.10 (Cohen, 1988) could be detected for comparisons involving two levels of the agent variable (e.g., anthropomorphized autonomous vehicle vs. autonomous vehicle) on the moral evaluations.

#### Materials and procedure

Materials and procedure—including the descriptions of the autonomous vehicle and the human driver—were identical to those of Experiment 1 with one exception. In addition to the two experimental conditions used in the first experiment (human driver and autonomous vehicle), we included a third condition with an anthropomorphized autonomous vehicle. This vehicle was introduced as a self-driving vehicle controlled by an intelligent driving system called “Alina.” Subsequently, the vehicle was only referred to by its name.

### Results

**Figure 3:**
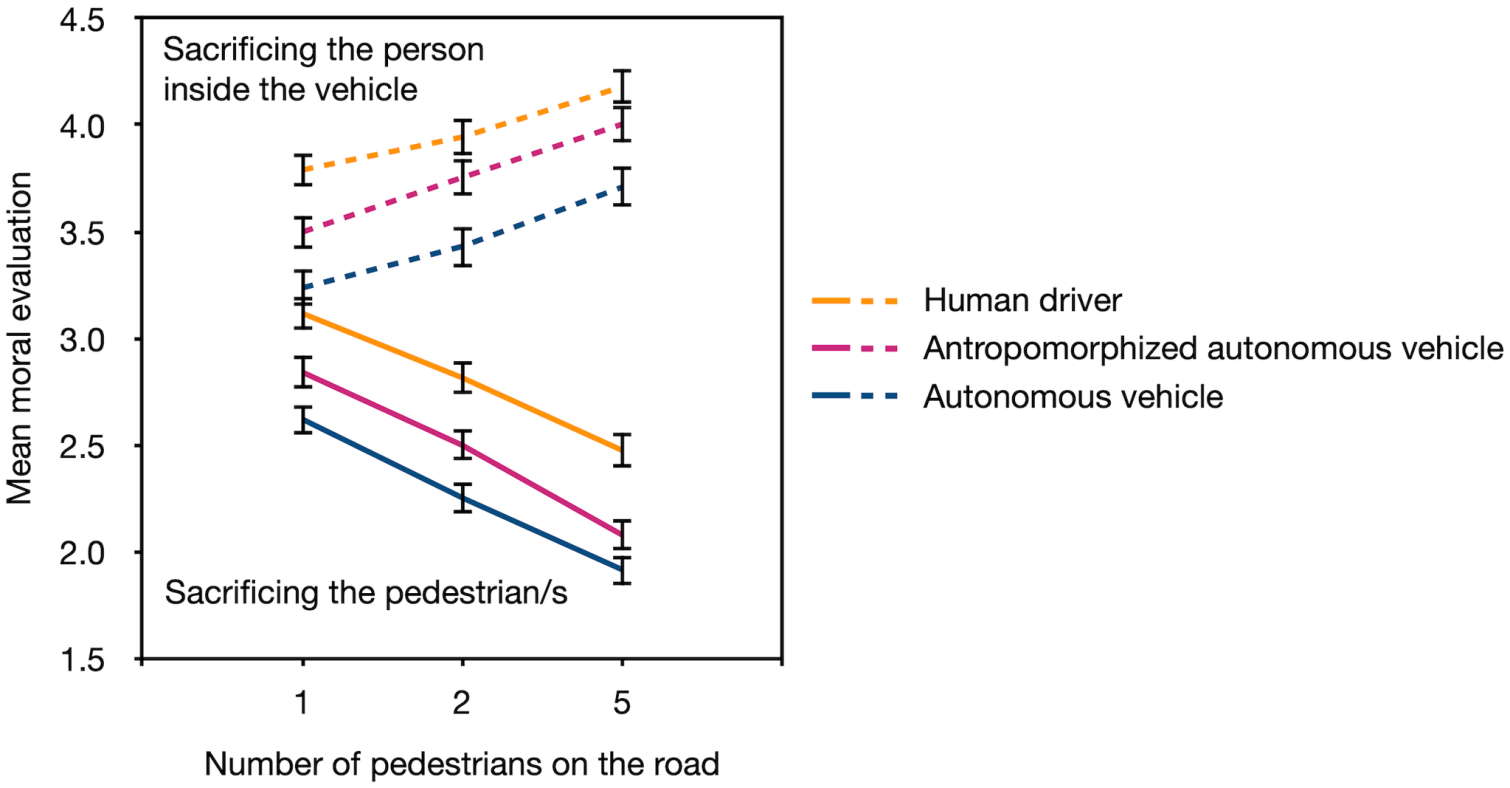
The mean moral evaluation of the actions (sacrificing the person inside the vehicle [dashed lines], sacrificing the pedestrian/s [solid lines]) as a function of the number of pedestrians on the road (1, 2, and 5) and the agent (human driver, anthropomorphized autonomous vehicle, autonomous vehicle). The moral-evaluation scale ranged from “very reprehensible” (1) to “very justifiable” (6). The error bars represent standard errors of the mean.

The data were analyzed in the same way as in Experiment 1. The mean moral evaluation of the actions as well as the standard errors of the means are depicted in Figure 3.

As in Experiment 1, there was a significant main effect of the agent, *F*(2,752) = 24.72, *p* < 0.001, *η_p_*^2^ = 0.06. Orthogonal Helmert contrasts showed that the actions of the human driver (*M* = 3.39, *SE* = 0.05) were evaluated more favorably from a moral perspective than the actions of both vehicle types together, *F*(1,752) = 37.76, *p* < 0.001, *η_p_*^2^ = 0.05, and that the actions of the anthropomorphized autonomous vehicle (*M* = 3.11, *SE* = 0.05) were evaluated more favorably than the actions of the autonomous vehicle (*M* = 2.86, *SE* = 0.06), *F*(1,752) = 11.35, *p* = 0.001, *η_p_*^2^ = 0.01. Sacrificing the person inside the vehicle (*M* = 3.72, *SE* = 0.05) was evaluated as more morally justifiable than sacrificing the pedestrian/s (*M* = 2.51, *SE* = 0.04), *F*(1,752) = 399.57, *p* < 0.001, *η_p_*^2^ = 0.35. The interaction between these two variables was not significant, *F*(2,752) = 0.30, *p* = 0.742, *η_p_*^2^ < 0.01.

The main effect of the number of pedestrians on the road was significant, *F*(2,751) = 28.18, *p* < 0.001, *η_p_*^2^ = 0.07 (*M* = 3.18, *SE* = 0.03, for one pedestrian; *M* = 3.11, *SE* = 0.03, for two pedestrians; *M* = 3.06, *SE* = 0.03, for five pedestrians). As in Experiment 1, the direction of this effect depended on the action that was taken, *F*(2,751) = 219.12, *p* < 0.001, *η_p_*^2^ = 0.37. An increase in the number of pedestrians led to a significant increase in the moral evaluation of the act of sacrificing the person inside the vehicle (*M* = 3.50, *SE* = 0.05, for one pedestrian; *M* = 3.71, *SE* = 0.05, for two pedestrians; *M* = 3.96, *SE* = 0.05, for five pedestrians; all comparisons *p* < 0.001) while it led to a significant decrease in the moral evaluation of sacrificing the pedestrian/s (*M* = 2.86, *SE* = 0.04, for one pedestrian; *M* = 2.52, *SE* = 0.04, for two pedestrians; *M* = 2.15, *SE* = 0.04, for five pedestrians; all comparisons *p* < 0.001). The effect of the number of pedestrians did not differ among the agents, *F*(4, 1,502) = 1.10, *p* = 0.353, *η_p_*^2^ < 0.01.

The three-way interaction was also not significant, *F*(4, 1,502) = 0.86, *p* = 0.485, *η_p_*^2^ < 0.01. When the person inside the vehicle was sacrificed, the action was evaluated as significantly more morally justifiable with an increasing number of pedestrians on the road for the human driver (*M* = 3.79, *SE* = 0.08, for one pedestrian; *M* = 3.94, *SE* = 0.08, for two pedestrians; *M* = 4.18, *SE* = 0.08, for five pedestrians; all comparisons *p* < 0.001), the anthropomorphized autonomous vehicle (*M* = 3.50, *SE* = 0.08, for one pedestrian; *M* = 3.75, *SE* = 0.08, for two pedestrians; *M* = 4.00, *SE* = 0.09, for five pedestrians; all comparisons *p* < 0.001), and the autonomous vehicle (*M* = 3.24, *SE* = 0.09, for one pedestrian; *M* = 3.43, *SE* = 0.09, for two pedestrians; *M* = 3.71, *SE* = 0.09, for five pedestrians; all comparisons *p* < 0.001). When the decision was to sacrifice the pedestrian/s, the opposite pattern was found for the human driver (*M* = 3.12, *SE* = 0.08, for one pedestrian; *M* = 2.81, *SE* = 0.07, for two pedestrians; *M* = 2.47, *SE* = 0.08, for five pedestrians; all comparisons *p* < 0.001), the anthropomorphized autonomous vehicle (*M* = 2.84, *SE* = 0.08, for one pedestrian; *M* = 2.50, *SE* = 0.07, for two pedestrians; *M* = 2.08, *SE* = 0.07, for five pedestrians, respectively; all comparisons *p* < 0.001), and the autonomous vehicle (*M* = 2.62, *SE* = 0.08, for one pedestrian; *M* = 2.25, *SE* = 0.07, for two pedestrians; *M* = 1.91, *SE* = 0.07, for five pedestrians; all comparisons *p* < 0.001).

### Discussion

Despite differences in the distributions of age and gender between the samples of Experiments 1 and 2, the overall pattern of results is very consistent. The global difference in the moral evaluation of the actions of the human driver and the autonomous vehicle observed in Experiment 1 was replicated in Experiment 2. Regardless of the type of action or the number of pedestrians on the road, the actions of the human driver were evaluated most favorably while the actions of the non-anthropomorphized autonomous vehicle were evaluated least favorably. This finding suggests that the actions of autonomous vehicles are more likely to be evaluated as morally reprehensible than the actions of human drivers. We were interested in whether it would be possible to narrow this evaluation gap by anthropomorphizing the autonomous vehicle. The anthropomorphization significantly reduced the evaluation gap between the human driver and the autonomous vehicle. The difference in the moral evaluation of the human driver and the autonomous vehicle was not completely eliminated but note that stronger manipulations (e. g., adding a human voice or other characteristics) may have stronger effects.

In addition, the results of Experiment 2 add to the evidence suggesting that utilitarian considerations are involved in the moral evaluations of both the human driver and the autonomous vehicles. Specifically, participants evaluated the actions of both the human driver and the autonomous vehicles more favorably if they were compatible with the utilitarian principle of saving more lives. Furthermore, if the life of one person inside the vehicle had to be weighed against the life of one pedestrian, participants evaluated the action that spared the life of the pedestrian more favorably than the action that spared the life of the person inside the vehicle. Despite the large sample size, we found no evidence that either of these effects differed as a function of whether the agent was a human driver or an autonomous vehicle. In terms of statistical tests, a statistically significant two-way interaction between agent and action and a statistically significant three-way interaction between agent, action, and number of pedestrians could have been interpreted as evidence of qualitative differences in the moral evaluation of the actions between human drivers and autonomous vehicles. These interactions were statistically significant but numerically small in Experiment 1 and clearly failed to replicate in Experiment 2 despite an increase in sample size which, in turn, resulted in an increased sensitivity to detect such effects. Overall, the evidence suggests that these interactions are negligeable.

## General discussion

The aim of the present study was to test whether differences in the moral evaluation of the actions of humans and machines can robustly be found in road-accident scenarios. The results provide support of the hypothesis that the actions of human drivers are judged more leniently than those of autonomous vehicles. Two main results support this hypothesis: (1) The results of both experiments consistently show that the actions of human drivers are judged as morally superior to those of autonomous vehicles. (2) Experiment 2 shows that anthropomorphizing the autonomous vehicle is effective in reducing the difference in the moral evaluations between the human driver and the autonomous vehicle. The results of Experiment 1 suggested that the more favorable moral evaluation of human drivers in comparison to autonomous vehicles may be modulated by utilitarian standards and self-sacrifice as suggested by previous theorizing (Malle et al., 2015; Sachdeva et al., 2015). However, both the two-way interaction between agent (human driver, autonomous vehicle) and action (sacrificing the person inside the vehicle, sacrificing the pedestrian/s) and the three-way interaction between agent, action, and number of pedestrians on the road (one pedestrian, two pedestrians, or five pedestrians) were only small in terms of sample effect sizes, and both interactions were not replicated in Experiment 2 despite the large sample size which provided favorable conditions for replicating the interactions if they were robust. The dominant pattern in both experiments was thus that the actions of human drivers were evaluated more favorably than those of autonomous vehicles. Descriptively, this pattern was present irrespective of whether the person inside the vehicle or the pedestrian/s on the road were sacrificed and irrespective of whether the action was in line with utilitarian standards or not. The more favorable evaluation of the actions of human drivers over autonomous vehicles that was observed in the present experiments is thus primarily due to a general bias rather than to a differential reliance on utilitarian principles or the specific moral admiration of the self-sacrifice of the human driver.

The present study thus helps to determine the nature of the differences in the moral evaluations of human drivers versus autonomous vehicles. The dominant pattern is that of a general bias toward judging the actions of human drivers as more morally permissible than those of autonomous vehicles. More research is necessary to understand the exact processes that underlie the bias toward the more favorable evaluation of the actions of human drivers in comparison to those of autonomous vehicles. One possibility is that of a moral-evaluation bias, that is, a general aversion against machines making life-and-death decisions (Bigman and Gray, 2018). This interpretation is in line with the finding of Young and Monroe (2019) that people blame autonomous vehicles more harshly than human drivers for their decisions in accident scenarios and with the finding of Gogoll and Uhl (2018) that people are reluctant to delegate a moral task to a machine. Possibly, this critical view of machines making moral decisions may stem from the fact that it is relatively easy for participants to put themselves in the human driver’s shoes and to imagine having experienced a conflict when making the decision which seems to be harder in case of a machine agent (Scheutz and Malle, 2021). Consequently, people might more easily justify (and potentially condone) the actions of a human agent compared to the actions of an autonomous vehicle. For example, participants might take into consideration that human drivers have to make spontaneous split-second decisions in critical traffic situations while autonomous vehicles are programmed in advance, the latter of which might make it easier to suspect bad intent. Further, the prospect of increased traffic safety—which is often linked to the introduction of autonomous vehicles—might also hint at higher expectations regarding the driving performance of autonomous vehicles compared to human drivers. There is, for example, evidence to suggest that the risks associated with autonomous vehicles are tolerated less than the risks associated with human drivers (Liu et al., 2020). A fatal accident might therefore represent an expectation violation in the case of an autonomous vehicle, which might contribute to the more negative moral evaluation of the actions of an autonomous vehicle compared to the actions of a human driver. Here it seems relevant that manipulations that make machines more human-like, for example by ascribing mental properties such as thoughts and feelings to them (Young and Monroe, 2019), reduce the evaluation gap between human drivers and autonomous vehicles. In line with this interpretation, Experiment 2 showed that anthropomorphizing the autonomous vehicle shifted the critical moral evaluation of the actions of the vehicle in the road-accident scenarios towards the more positive moral evaluations of the same actions performed by a human driver. This finding is in line with the observation that anthropomorphizing can positively affect the perception of a machine agent (e.g., Gong, 2008; Lee et al., 2015; Niu et al., 2018). A limitation of the present study is that the specific attributes that are responsible for the more human-like judgement of the anthropomorphized vehicle are yet to be determined. The autonomous vehicle was assigned a name and described as an intelligent driving system. Humans and machines differ in a number of characteristics such as their perceived agency and the mental capacities (perception, emotion, learning, and thinking) which people might attribute to them. These attributions may change as the technology and people’s experience with autonomous driving systems and computer algorithms evolves. We do not know how exactly the attribution of humanlike properties to the autonomous vehicles have influenced participants’ assumptions about these characteristics and, consequently, the exact cognitions that underlie the observed moral-evaluation bias. Understanding these underlying processes is an important goal for future studies.

When interpreting the present results, it should be considered that participants were asked to evaluate abstract road-accident scenarios fashioned after moral dilemmas which is a common research paradigm to examine moral evaluations and decisions (for other studies employing abstract scenarios see, e.g., Bonnefon et al., 2016; Awad et al., 2018; Frank et al., 2019). Moral dilemmas are useful to identify factors of a scenario that are relevant for its evaluation (e.g., Hauser et al., 2007; Keeling, 2020), to probe different ethical principles or theories and to investigate moral intuitions and moral decision making (e.g., Hauser et al., 2007; Cushman and Greene, 2012; Goodall, 2016; Wolkenstein, 2018). Abstract scenarios obviously fall short of real-life accidents experienced first-hand but they bear resemblance to newspaper reports on accidents. Newspaper reports probably are associated with low levels of immersion as they primarily describe the accident itself and perhaps the accident’s causes and consequences. In that sense abstract scenarios seem suitable for investigating how the public will react to accidents with autonomous vehicles they read about in the newspaper. This seems quite relevant given that it is more likely for the majority of people to learn about accidents from newspaper reports than by witnessing, or being directly involved in, an accident. Nevertheless, it has to be counted among the limitations of the present study that we cannot draw conclusions about situations in which there is a more direct involvement in the accidents. Furthermore, the conclusions of the present study are necessarily limited by the specific conditions that were included in the present experiments. While the involvement of utilitarian standards and the presence or absence of self-sacrifice were varied, moral evaluations may depend on many other factors such as the violation of rules and obligations or social prejudices and biases (e.g., Awad et al., 2018; Frank et al., 2019). The present study thus cannot shed light on the degree to which the moral evaluations of humans and machines are influenced by these factors.

The presents study’s aim was neither to develop guidelines for programming autonomous vehicles (e.g., Wolkenstein, 2018) nor to determine whether autonomous vehicles or other machines can be regarded as moral agents (for some points of view see, e.g., Li et al., 2016; Gogoll and Müller, 2017; Bonnefon et al., 2019; Scheutz and Malle, 2021) and in how far concepts such as responsibility, liability, or blame can or should be assigned to machines. We focused on the moral evaluation of actions in critical traffic situations as this might represent a first step in understanding the public’s reaction to accidents with autonomous vehicles. The evaluation of the agent itself or questions of blame and responsibility are separate issues. Investigating how actions of different agents are perceived in critical traffic situations is important in order to anticipate potential problems regarding the acceptance of autonomous vehicles. In this respect, the perception and opinion of ordinary people is especially relevant as they have to accept the technology (Malle et al., 2019). Gogoll and Uhl (2018) have argued that a disliking of autonomous vehicles making moral decisions has the potential to slow down automation in driving. Considering and openly addressing differences in the moral evaluation of human drivers and autonomous vehicles could thus be beneficial for the introduction and the success of autonomous driving technologies.

In conclusion, the present study contributes to our understanding of how moral norms are applied to machine agents. People have a bias toward judging actions of human drivers as morally superior to identical actions (with identical consequences) of autonomous vehicles. Accidents resembling moral dilemmas might be rare but they are emotionally salient (Bonnefon et al., 2016) and there is evidence to suggest that moral dilemmas are regarded as an important challenge for autonomous vehicles (Gill, 2021). Moral decisions—which include decisions about how to distribute harm in accident situations—have the potential to affect the perception of autonomous vehicles via media coverage of accidents (e.g., Anania et al., 2018). At least during the early introduction phases, a strong media attention to accidents involving autonomous vehicles seems likely (Shariff et al., 2017). A more negative moral evaluation of the actions of autonomous vehicles in comparison to those of human drivers may have negative effects on the acceptance of autonomous driving technologies (see also Gogoll and Uhl, 2018). Therefore, it seems relevant to search for interventions that may decrease the differential moral evaluations of human drivers and autonomous vehicles. The results of Experiment 2 suggest that anthropomorphizing autonomous vehicles can reduce the action evaluation gap between autonomous vehicles and human drivers. Thus, assigning human characteristics to autonomous vehicles might represent a promising intervention for transferring some of the leniency people display towards human drivers to autonomous vehicles.

## Data availability statement

The datasets presented in this study can be found in online repositories. The names of the repository/repositories and accession number(s) can be found at: https://osf.io/3xamb/.

## Ethics statement

Ethical review and approval was not required for the study on human participants in accordance with the local legislation and institutional requirements. The patients/participants provided their written informed consent to participate in this study.

## Author contributions

MM, AB, and RB participated in planning the research, designing the experiments, analyzing the collected data, and interpreting the results. MM created the materials, collected the data, and wrote the initial manuscript. AB and RB provided feedback and contributed to the manuscript. All authors contributed to the article and approved the submitted version.

## Funding

The publication of this article is supported by the open access fund of Heinrich Heine University Düsseldorf.

## Conflict of interest

The authors declare that the research was conducted in the absence of any commercial or financial relationships that could be construed as a potential conflict of interest.

## Publisher’s note

All claims expressed in this article are solely those of the authors and do not necessarily represent those of their affiliated organizations, or those of the publisher, the editors and the reviewers. Any product that may be evaluated in this article, or claim that may be made by its manufacturer, is not guaranteed or endorsed by the publisher.
